# Multidrug-resistant Pseudomonas aeruginosa producing PER-1 extended-spectrum serine-beta-lactamase and VIM-2 metallo-beta-lactamase.

**DOI:** 10.3201/eid0705.010528

**Published:** 2001

**Authors:** J D Docquier, F Luzzaro, G Amicosante, A Toniolo, G M Rossolini


6. Goldwasser RA, Steiman Y, Klingberg W, Swartz TA, Klingberg MA. The isolation of strains of rickettsiae of the spotted fever group in Israel and their differentiation from other members of the group by immunofluorescence methods. Scand J Infect Dis 1974;6:53-62.
7. Beati L, Finidori JP, Gilot B, Raoult D. Comparison of serologic typing, sodium dodecyl sulfate-polyacrylamide gel electrophoresis protein analysis, and genetic restriction fragment length polymorphism analysis for identification of rickettsiae: characterization of two new rickettsial strains. J Clin Microbiol 1992;30:1922-30.
8. Roux V, Rydkina E, Eremeeva M, Raoult D. Citrate synthase gene comparison, a new tool for phylogenetic analysis, and its application for the rickettsiae. Int J Syst Bacteriol 1997;47:252-61.
9. Fournier PE, Roux V, Raoult D. Phylogenetic analysis of spotted fever group rickettsiae by study of the outer surface protein rOmpA. Int J Syst Bacteriol 1998;48:839-49.
10. Roux V, Raoult D. Phylogenetic analysis of members of the genus *Rickettsia* using the gene encoding the outer-membrane protein rOmpB. Int J Syst Evol Microbiol 2000;50:1449-55.
11. Lascola B, Raoult D. Laboratory diagnosis of rickettsioses: current approaches to diagnosis of old and new rickettsial diseases. J Clin Microbiol 1997;35:2715-27.

---

### Multidrug-Resistant *Pseudomonas aeruginosa* Producing PER-1 Extended-Spectrum Serine-ß-Lactamase and VIM-2 Metallo-ß-Lactamase

**To the Editor:** In *Pseudomonas aeruginosa*, secondary beta-lactamases with extended substrate specificity can be responsible for acquired resistance to the most powerful antipseudomonal beta-lactams, such as expanded-spectrum cephalosporins and carbapenems (1). A number of these enzymes have been described, including extended-spectrum serine-beta-lactamases (ESBLs) of groups 2be and 2d (e.g., PER-1 and various OXA-type enzymes) (2,3) and metallo-beta-lactamases of group 3 (e.g., IMP-1 and the recently described VIM-1 and VIM-2 enzymes) (2,4,5). The secondary ESBLs can degrade penicillins, expanded-spectrum cephalosporins, and monobactams (but not carbapenems) and are often susceptible to serine-beta-lactamase inhibitors (1-3). The secondary metallo-beta-lactamases, on the other hand, are notable for their carbapenemase activity and can degrade virtually all beta-lactams except monobactams, while being resistant to the currently available inhibitors (1,2,5,6).

On March 2000, a multidrug-resistant *P. aeruginosa* (isolate VA-182/00) was isolated in pure culture from a bronchial washing of a 58-year-old patient with multiple myeloma. The patient had been admitted 15 days earlier to the Varese University Hospital with a diagnosis of pneumonia and had been treated with ciprofloxacin (0.5 g twice a day) plus piperacillin (2 g three times a day) for 12 days, and then with imipenem/cilastatin (0.5 g three times a day). No cultures of respiratory tract specimens were done earlier in hospitalization. Multiple myeloma had been diagnosed in 1997, and the patient had been treated with multiple cycles of antiproliferative chemotherapy and had received autologous peripheral blood stem cell transplantation. According to clinical records, *P. aeruginosa* had not been isolated previously during this patient's protracted illness. In vitro susceptibility testing showed that the *P. aeruginosa* isolate was resistant to mezlocillin, ceftazidime, cefepime, aztreonam, imipenem, meropenem, gentamicin, tobramycin, netilmicyn (MICs, >128 µg/mL), amikacin (MIC, 64 µg/mL), ciprofloxacin and levofloxacin (MICs, >32 µg/mL). Only piperacillin and piperacillin/tazobactam had MIC values slightly lower than the breakpoints for resistance (64 µg/mL and 48/4 µg/mL, respectively), although–considering the normal MICs of piperacillin for susceptible *P. aeruginosa* (2-8 µg/mL)–it was evident that the isolate also had considerable biological resistance to these drugs. A double disk-diffusion test, carried out with standard disks placed 20 mm apart (center-to-center), showed synergy between clavulanate and aztreonam. The treatment was changed to piperacillin/tazobactam (4 g four times a day), and a slow recovery ensued over a 30-day period. The patient died 3 months later following a relapse of the underlying malignancy.

The unusually high carbapenem MICs exhibited by VA-182/00 suggested production of a secondary metallo-beta-lactamase, while the synergy between clavulanate and aztreonam suggested production of a secondary serine ESBL. A crude extract of that isolate, assayed spectrophotometrically (7), exhibited imipenem-hydrolyzing activity (94 nmol/min/mg protein, inhibited by EDTA) as well as aztreonam-hydrolyzing activity (11 nmol/min/mg protein, resistant to EDTA). Analytic isoelectric focusing (IEF) of the extract, followed by development with the nitrocefin chromogenic substrate (7), showed three bands of beta-lactamase activity of pIs 5.4, 5.6, and 6.3, suggesting the presence of at least three different secondary enzymes. A colony-blot hybridization with probes for the $bla_{IMP}$, $bla_{VIM}$, and $bla_{PER}$ resistance genes (all of which have been previously detected in *P. aeruginosa* clinical isolates from the same hospital [8,9; Luzzaro F, unpub. data]) yielded positive results with both the $bla_{VIM}$ and the $bla_{PER}$ probes. Amplification of the resistance genes by polymerase chain reaction (PCR) with primers VIM/DIA-f (5'-CAgATTgCCgATggTgTTTgg) and VIM/DIA-r (5'-AggTgggCCATTCAgCCAgA) for $bla_{VIM}$ genes (4,5) and BLAPER-f (5'-gggACA(g/A)TC(g/C)(g/T)ATgAATgTCA) and BLAPER-r (5'-ggg(C/T)(g/C)gCTTAgATAgTgCTgAT) for $bla_{PER}$ genes (9), yielded amplicons of the expected sizes (522 and 966 bp, respectively). Direct amplicon sequencing identified the two beta-lactamase determinants as $bla_{VIM-2}$ (5) and $bla_{PER-1}$ (10), respectively, a finding consistent with the pIs 5.6 and 5.4 beta-lactamase bands detected in IEF (3,5). Conjugative transfer of the resistance determinants to *Escherichia coli* proved unsuccessful. In a Southern blot analysis of total undigested DNA from VA-182/00, both the $bla_{VIM}$ and $bla_{PER}$ probes apparently hybridized to the chromosomal DNA band; no plasmid bands recognized by either probe were detected. A PCR experiment with primers OXA10-f (5'-ggAACAAAgAgTTCTCTgCC) and OXA105-r (5'-TTAgCCACCAATgATgCC(C/T)TC), suitable for amplification of $bla_{OXA}$ genes of the OXA-10 group, did not yield an amplicon of the expected size (719 bp), suggesting that the pI 6.3 beta-lactamase band detected by IEF did not correspond to an enzyme of this group.

This is the first observation of a *P. aeruginosa* clinical isolate simultaneously producing a secondary PER-1 ESBL and a secondary metallo-beta-lactamase. The finding, observed in a hospital where both the resistance genes

(*bla*$_{PER-1}$ and *bla*$_{VIM-2}$) had been detected separately among clinical isolates, underscores the possibility of the emergence of new threatening combinations of resistance determinants among nosocomial pathogens. In fact, the recruitment of similar resistance determinants within a single *P. aeruginosa* strain can determine a resistance phenotype to virtually all the available antipseudomonal beta-lactams, an occurrence that can be particularly dramatic when, as in the present case, resistance to beta-lactams is associated with resistance against aminoglycosides and fluoroquinolones. In this case, only piperacillin (which appears to be a relatively poor substrate for both enzymes [3,5]) retained moderate activity in vitro and, administered at high dosage in combination with tazobactam, was apparently effective in vivo. Should a similar resistance phenotype disseminate, it might have strategic implications for the development of new beta-lactamase inhibitors and for selection of beta-lactam compounds to associate wth inhibitors.

### Acknowledgments

This work was supported in part by grant no. FMRX-CT98-0232 from the European Training and Mobility of Researchers Network on metallo-beta-lactamases.

**Jean-Denis Docquier,* Francesco Luzzaro,† Gianfranco Amicosante,‡ Antonio Toniolo,† and Gian Maria Rossolini***

*Dipartimento di Biologia Molecolare, Sezione di Microbiologia, Università di Siena, Siena, Italy; †Laboratorio di Microbiologia, Ospedale di Circolo e Università dell'Insubria, Varese, Italy; and ‡Dipartimento di Scienze e Tecnologie Biomediche, Università di L'Aquila, L'Aquila, Italy

### References

1. Livermore DM. Beta-Lactamases in laboratory and clinical resistance. Clin Microbiol Rev 1995;8:557-84.
2. Bush K, Jacoby GA, Medeiros AA. A functional classification scheme for beta-lactamases and its correlation with molecular structure. Antimicrob Agents Chemother 1995;39:1211-33.
3. Nordmann P, Ronco E, Naas T, Duport C, Michel-Briand Y, Labia R. Characterization of a novel beta-lactamase from *Pseudomonas aeruginosa*. Antimicrob Agents Chemother 1993;37:962-9.
4. Lauretti L, Riccio ML, Mazzariol A, Cornaglia G, Amicosante G, Fontana R, et al. Cloning and characterization of bla$_{VIM}$, a new integron-borne metallo-beta-lactamase gene from a *Pseudomonas aeruginosa* clinical isolate. Antimicrob Agents Chemother 1999;43:1584-90.
5. Poirel L, Naas T, Nicholas D, Collet L, Bellais S, Cavallo JD, et al. Characterization of VIM-2, a carbapenem-hydrolyzing metallo-beta-lactamase and its plasmid- and integron-borne gene from a *Pseudomonas aeruginosa* clinical isolate in France. Antimicrob Agents Chemother 2000;44:891-97.
6. Franceschini N, Caravelli B, Docquier JD, Galleni M, Frere JM, Amicosante G, et al. Purification and biochemical characterization of the VIM-1 metallo-beta-lactamase. Antimicrob Agents Chemother 2000;44:3003-7.
7. Livermore DM, Williams JD. Beta-lactams: mode of action and mechanisms of bacterial resistance. In: Lorian V, editor. Antibiotics in Laboratory Medicine. 4th ed. Baltimore: William & Wilkins; 1996. p. 502-78.
8. Luzzaro F, Mantengoli E, Perilli M, Lombardi G, Orlandi V, Orsatti A, et al. Dynamics of a nosocomial outbreak of multidrug-resistant *Pseudomonas aeruginosa* producing the PER-1 extended-spectrum beta-lactamase. J Clin Microbiol 2001;39:1865-70.
9. Pereira M, Perilli M, Mantengoli E, Luzzaro F, Toniolo A, Rossolini GM, et al. PER-1 extended-spectrum beta-lactamase production in an *Alcaligenes faecalis* clinical isolate resistant to expanded-spectrum cephalosporins and monobactams from a hospital in Northern Italy. Microb Drug Resist 2000;6:85-90.
10. Nordmann P, Naas T. Sequence analysis of PER-1 extended-spectrum beta-lactamase from *Pseudomonas aeruginosa* and comparison with class A beta-lactamases. Antimicrob Agents Chemother 1994;38:104-14.

### Jamestown Canyon Virus: Seroprevalence in Connecticut

**To the Editor:** *Jamestown Canyon virus* (JCV), a member of the California serogroup, has a wide geographic distribution throughout much of temperate North America. It causes mild febrile illness and, rarely, aseptic meningitis or primary encephalitis (1). JCV has been isolated from mosquitoes each year that surveys have been done in Connecticut, and 28 positive pools from 10 mosquito species were found during 2000 (T. Andreadis, pers. commun.). In contrast, only 14 positive mosquito pools were found to contain *West Nile virus* (WNV), which has recently been introduced into Connecticut (2). JCV has been isolated from *Aedes* mosquitoes in Connecticut, and serologic evidence suggests it is widespread in deer (3,4). No recent seroprevalence surveys have been done in Connecticut, nor have any human cases of infection or disease due to JCV been documented.

We report the results of two seroprevalence surveys done with standard indirect fluorescent assays (IFA) to detect immunoglobulin G antibodies to JCV. One survey examined 1,086 sera collected in 1990 from blood donors. The second survey examined 1,016 sera submitted to the Connecticut State Public Health Laboratory in 1995.

The IFA used JCV-infected baby hamster kidney cells (BHK-21). Infected and uninfected cell suspensions were air dried and fixed onto Teflon-coated, 12-well slides. Prepared slides were stored at -70°C. Sera were tested at a minimum dilution of 1:16. After incubation and washing of the fluorescein-conjugated counterstain, slides were dried and examined by fluorescent microscope (American Optical, Buffalo, NY). The positive human control serum was designated as the 4+ baseline with which the test sera were compared. Selected sera were tested by a serum dilution plaque reduction neutralization test (PRNT) assay with JCV, *La Crosse virus*, and trivittatus virus.

Of the 1,086 sera collected from blood donors in 1990, 164 (15%) were positive by IFA at a minimum dilution of 1:16. Because IFA screening procedures are known to have poor specificity, a subset of 39 IFA-positive and 5 IFA-negative sera was tested by PRNT. None of the IFA-negative sera were positive, while 26 (67%) of the 39 IFA-positive sera were positive for JCV antibodies. Extrapolating the PRNT results to the 164 IFA-positive sera yields an overall positivity rate of 10.1%.

The second serosurvey, performed on 1,016 sera collected in 1995 from apparently healthy patients requesting immune status testing to viruses such as *Varicella zoster* or measles, had 57 IFA-positive specimens. Extrapolating addi-

